# Item

**Published:** 1902-03

**Authors:** 


### ITEM.

Messrs. Parke, Davis & Company, of Detroit, have adopted the plan of encouraging their employes to become stockholders —a policy inspired by philanthropic purposes. The company proposes to issue 4,000 shares of its capital stock nd permit the oldest among its employes, especially those in important positions as managers, superintendents and foremen, to purchase this new stock at $55 a share. The present market value of the stock is $70 a share, and face value $25 a share. This is one of the institutions of which Detroit is very proud. From a small beginning in the early '70's the business has developed into world-wide proportions.

